# Crosstalk of *KCNH1* and *KCNH5* gain-of-function mutations leading to epilepsy and neurodevelopmental disorders

**DOI:** 10.1186/s13041-026-01279-1

**Published:** 2026-02-08

**Authors:** Alisa Bernert, Philipp Rühl, Roland Schönherr, Stefan H. Heinemann

**Affiliations:** Department of Biophysics, Center for Molecular Biomedicine, Friedrich Schiller University Jena and Jena University Hospital, Hans-Knöll-Straße 2, 07745 Jena, Germany

**Keywords:** Kv10.1, Kv10.2, Ether à go-go, K^+^ channel, Temple-Baraitser syndrome, Zimmermann-Laband syndrome, Epilepsy, Heteromerization

## Abstract

Voltage-gated K^+^ (Kv) channels are tetrameric complexes of proteins encoded by *KCN* genes. Gain-of-function (GoF) mutations in *KCNH1* (Kv10.1, hEAG1) and *KCNH5* (Kv10.2, hEAG2) give rise to developmental disorders, intellectual disability, and epilepsy. Currently, clinical symptoms are not straightforwardly associated with functional properties of mutated channels. Here we investigated how members of the *KCNH* subfamily are affected by heteromerization with mutant Kv10.1 or Kv10.2 protein subunits. The *de novo* variant Kv10.1-G496E, which leads to impaired neurodevelopment and epilepsy, was expressed alone or with other wild-type subunits in HEK293T cells and characterized using whole-cell patch clamp. While Kv10.1-G496E alone did not yield functional K^+^ channels, coexpression with Kv10.1 or Kv10.2 shifted the half-maximum voltage of activation in the hyperpolarizing direction. Likewise, the homologous mutation Kv10.2-G465E did not yield functional channels but also induced GoF upon coexpression with wild-type Kv10.1 or Kv10.2. By contrast, the mutants did not affect the function of Kv11.1 (*KCNH2*, hERG1) channels. To infer the relevance of Kv10 GoF mutations under physiological conditions, we used the fluorescent genetically encoded voltage indicator mK2-rEstus and found that both, Kv10.1 and Kv10.2, hyperpolarized HEK293T cells, and that coexpression of the GoF mutants augmented this hyperpolarization. Our findings imply that interpretation of clinical symptoms related to Kv10 GoF mutations requires considering the functional crosstalk with Kv10.1 and Kv10.2 subunits, which are both expressed in glutamatergic neurons in cortical Layers III and IV.

**Supplementary Information:**

The online version contains supplementary material available at 10.1186/s13041-026-01279-1.

## Introduction

Voltage-gated potassium ion (Kv) channels are tetrameric protein complexes of protein subunits encoded by *KCN* genes. In contrast to voltage-gated Na^+^ channels or Ca^2+^ channels, the tetrameric assembly of Kv channels from individual subunits bears the potential to form heteromeric complexes and thus to broaden the spectrum of functional channel types [1]. The formation of functional heteromers is common among closely related proteins encoded by genes of the same subfamily [2]. Heteromer formation in K^+^ channels not only widens their functional spectrum but can also be relevant to channelopathies, diseases caused by mutated channel genes. For example, in Andersen-Tawil syndrome, caused by mutant Kir2.1 channels [3], a large phenotypic variability among patients has been attributed, at least in part, to heteromerization of mutated dominant negative Kir2.1 channel subunits with Kir2.2 or Kir2.3 subunits in cardiac or skeletal muscle [4]. Similarly, owing to heteromerization of wild-type and mutant subunits, Ca^2+^ - and voltage-activated K^+^ channels of large conductance (*KCNMA1*) display a wide range of functional features [5].

The ether à go-go family of Kv channels (*KCNH*) comprises three distinct subfamilies: Kv10 (EAG: Kv10.1/*KCNH1*, Kv10.2/*KCNH5*), Kv11 (ERG: Kv11.1/*KCNH2*, Kv11.2/*KCNH6*, Kv11.3/*KCNH7*), and Kv12 (ELK: Kv12.1/*KCNH8*, Kv12.2/*KCNH3*, Kv12.3/*KCNH4*) [6, 7]. For all three subfamilies, intragroup heteromerization is possible, as demonstrated in coexpression experiments. Evaluation of dominant-negative mutants of Kv11.1 and Kv11.2 revealed that all three Kv11 isoforms can coassemble, while members of the related subfamilies Kv10 (Kv10.1) and Kv12 (Kv12.1) were not affected by the dominant-negative Kv11 mutants [8]. Studies on Erg K^+^ currents in mouse Purkinje neurons provided evidence for endogenous functional Erg1/Erg3 heteromers in situ [9]. The strategy of dominant-negative mutants in heterologous expression systems was also applied to the Kv10 subfamily members, confirming heteromer formation between Kv10.1 and Kv10.2, while intergroup heteromerization with less-related Kv1.5 channels was excluded [10]. For the Kv12 subfamily, intragroup heteromers were shown for all three members, while dominant-negative Kv12 isoforms did not functionally interact with Kv10.2 or Kv11.3 channels in the *Xenopus* oocyte expression system [11].

Given that intragroup heteromerization is possible in all three *KCNH* subfamilies, any dominant disease-related mutation in one gene may have the potential to influence more than one type of channel, provided subunits of the same group are expressed in the same cell type. For Kv10.1 and Kv10.2, several disease-related and dominant gain of function (GoF) mutations have been described. Point mutations in *KCNH1* (encoding Kv10.1) cause the closely related Zimmermann-Laband syndrome (ZLS) [12] and Temple-Baraitser syndrome (TBS) [13]. ZLS and TBS are characterized by intellectual disability, various kinds of dysmorphisms of face, phalanges or gingiva, and a high probability of epileptic seizures. The clinical comparison of the described symptoms reveals a wide phenotypic variability and some degree of overlap between both syndromes, with some cases that fall outside the classical ZLS/TBS diagnosis yet lie within the wider phenotypic spectrum [14]. The first described disease-causing mutation in *KCNH5* (encoding Kv10.2) was the dominant GoF mutation R327H, which causes a strong hyperpolarizing shift in the voltage dependence of activation [15, 16]. More recent screens of patients with developmental and epileptic encephalopathies have identified more *KCNH5* mutations [17, 18]. Developmental concerns affecting language or cognitive outcomes were observed in some but not all of these patients, while no significant dysmorphic features were described [17]. Overall, besides dysmorphism, epileptic encephalopathy is now recognized as part of the diverse spectrum of Kv10-related symptoms [19]. Moreover, *KCNH5* belongs to the risk genes for autism spectrum disorders (ASD) [18, 20].

Kv10.1 and Kv10.2 display unique functional features; for example, their speed of voltage-dependent activation is slowed down by extracellular Mg^2+^ and hyperpolarization [21]. The channels are inhibited by intracellular Ca^2+^/calmodulin [22], and the channel activity is diminished at increased levels of membrane phosphatidylinositol 4,5-bisphosphate (PIP_2_) [23]. Both, *KCNH1* and *KCNH5*, are expressed in the brain [24], but their physiological functions and pathophysiological mechanisms by which GoF mutations cause the respective symptoms are still elusive. An involvement in early developmental stages aligns with the documented upregulation of *KCNH1* and *KCNH5* in various cancer entities [25].

Attempts to correlate genotype and clinical phenotypes of Kv10.1 and Kv10.2 mutations typically address the two genes as clearly separate entities. However, the possibility of forming heteromeric channels within this subfamily may contribute to the wide and rather unpredictable patterns of clinical outcomes. If both genes have overlapping expression patterns in the adult organism or during embryonic development, a functional crosstalk of dominant mutants between both channel types should be considered. Here we chose a coexpression strategy in HEK293T cells to address the question of whether mutants of Kv10.1 affect coexpressed Kv10.2 subunits and vice versa. For this purpose, we functionally characterized the *KCNH1* mutant Kv10.1-G496E (Fig. S1), which has been reported to cause *inter alia* severe intellectual disability, developmental delay, and epilepsy [26]. We furthermore investigate the physiological outcome of channel expression in HEK293T cells as well as of the mixed coexpression of wild-type and mutant variants by noninvasively measuring their impact on the resting membrane potential.

## Materials and methods

### Expression plasmids, cell culture and transfection

The coding sequences of human Kv10.1 (short splice form, hEAG1a, *KCNH1*, Acc. no. NM_002238), Kv10.1_long_ (long splice form, hEAG1b, *KCNH1*, Acc. no. NM_172362), Kv10.2 (hEAG2, *KCNH5*, Acc. no AF418206), and Kv11.1 (hERG1, *KCNH2*, Acc. no. NM_000238) were expressed in HEK293T cells as described previously [27]. Mutations were generated using PCR-based methods followed by DNA sequencing. The numbering Kv10.1-G496E is based on the long splice variant of *KCNH1* (hEAG1b), as this is typically found in mutant databases. However, the majority of the functional evaluation was performed with the short splice form (hEAG1a), which would render the mutation site Kv10.1-G469E; for consistency, we term it Kv10.1-G496E or Kv10.1_long_-G496E if the long variant is referred to. All channel sequences were inserted into plasmids containing a CMV promoter.

Human embryonic kidney 293T (HEK293T, CAMR; Porton Down, Salisbury, UK) cells were cultured in a medium consisting of a 1:1 mixture of Dulbecco’s Modified Eagle’s Medium and Nutrient Mixture F-12 (Thermo Fisher Scientific, Waltham, Massachusetts, USA), supplemented with 10% fetal bovine serum. The cells were maintained in a humidified incubator at 37 °C with 5% CO_2_, and subsequently transfected with the channel-coding plasmids using ROTI®Fect (Carl Roth, Karlsruhe, Germany). Visualization of transfected cells was achieved by coexpressing CD8+ and subsequent incubation of the cells with anti-CD8-coated beads. Plasmids coding for wild-type channels were coexpressed with *KCNH1*-G496E or *KCNH5*-G465E at an approximate weight ratio of 1:1 or, as indicated, 1:3. Mock transfection of HEK293T cells was performed with the expression of CD8+ alone.

### Electrophysiological recordings

Currents mediated by the Kv channels were measured in the whole-cell configuration of the patch-clamp method using an EPC10 amplifier (HEKA Elektronik, Lambrecht, Germany) and PATCHMASTER NEXT data acquisition software (Multi Channel Systems MCS GmbH, Reutlingen, Germany). Pipettes were fabricated from borosilicate glass with filament, tips coated with dental wax and fire-polished to yield resistances between 1.2 and 2.5 MΩ. Current data were sampled at a rate of 10 kHz with a 3-kHz low-pass anti-aliasing filter. Only cells with series resistance below 5 MΩ were accepted; the series resistance was corrected electronically up to 75%. For display and analysis purposes, current traces were further filtered with a Gaussian characteristic and a cutoff frequency of 1 kHz. The holding membrane voltage was −100 mV or, as indicated, −120 mV. Data acquisition started after > 10 min of equilibration of the cell in the whole-cell configuration. For Kv10 channels, linear leak currents were subtracted offline; a p/4 leak-subtraction protocol was applied for Kv11.1 channels.

For the recording of Kv10 currents, the pipette (intracellular) solution contained (in mM): 130 KCl, 10 EGTA, 10 HEPES, pH 7.4 (NMG); the extracellular (bath) solution contained: 140 KCl, 1.5 CaCl_2_, 1.0 MgCl_2_, 10 HEPES, pH 7.4 (NMG), thus yielding a reversal potential for K^+^ of 2 mV in order to visualize channel opening in the range of typical resting voltages. The extracellular solution for Kv11.1 currents was (in mM): 145 NaCl, 5 KCl, 1.5 CaCl_2_, 1.0 MgCl_2_, 10 HEPES, pH 7.4 (NaOH).

*Current-voltage relationships*: Currents were elicited with 5-s depolarizing steps from −120 to 30 mV in increments of 10 mV, returning to −120 mV. The resulting mean currents of wild-type channels during 100 ms before the end of the depolarizing segment *I*(*V*) were fitted assuming a linear single-channel characteristic and *n* = 1 activation gates according to Hodgkin and Huxley:

1 $$ I(V) = \Gamma \left( {V - E_{{rev}} } \right)\;\left( {1 + e^{{ - {{\left( {V - V_{n} } \right)} \mathord{\left/ {\vphantom {{\left( {V - V_{n} } \right)} {k_{n} }}} \right. \kern-\nulldelimiterspace} {k_{n} }}}} } \right)^{{ - 1}} $$

with the total conductance Γ, the reversal potential *E*_rev_, the half-maximal voltage of gate activation *V*_n_, and the slope factor *k*_n_ characterizing the voltage dependence. While this approach does not always result in the best data descriptions—because more than one subunit may contribute to the voltage dependence of the observed open probability—we used this description with only one activation gate to obtain *V*_n_ and *k*_n_ values that can be directly compared under the experimental conditions examined.

For the description of current-voltage data originating from the coexpression of Kv10.1 or Kv10.2 with Kv10.1-G496E, we made several assumptions: (i) the major impact of the mutation G496E is to left-shift the *V*_n_ value; (ii) the hypothetical *V*_n_ value for a channel formed from four G496E subunits (“hypothetical” because this assembly does not result in functional channels) is *V*_ns_; (iii) for simplicity we assume that the voltage sensitivity, expressed by *k*_n_, is not affected by the mutation; (iv) the IV relationship of currents arising from coexpression accounts for the binomially distributed channel composition of wild-type and mutant subunits. We assumed that there is a linear relationship between the number of mutant subunits and the resulting *V*_n_ value. The following ratios of wild type to mutant should yield functional channels: 4:0 (*V*_n_); 3:1 (*V*_n_ + 1/4 * (*V*_ns_-*V*_n_)); 2:2 (*V*_n_ + 2/4 * (*V*_ns_-*V*_n_)); 1:3 (*V*_n_ + 3/4 * (*V*_ns_-*V*_n_)); 0:4 (no current). The binomial weighting factors depend on the availability and/or integration propensity of the respective subunits, *p*_s_. Thus, an IV data fit for coexpression will yield *V*_ns_ and *p*_s_ with *V*_n_ and *k*_n_ already known from the control experiments with wild-type channels.

For Kv11.1 channels, which undergo substantial inactivation, the voltage dependence of channel activation was measured by determining the tail currents at −120 mV, following the depolarizing segment. The resulting voltage dependence of the tail current (*I*_tail_) was described as:

2 $$ \frac{{I_{{tail}} (V)}}{{I_{{tail}} (30\,mV)}} = r_{{ - \infty }} + \frac{{r_{\infty } - r_{{ - \infty }} }}{{1 + e^{{ - {{\left( {V - V_{n} } \right)} \mathord{\left/ {\vphantom {{\left( {V - V_{n} } \right)} {k_{n} }}} \right. \kern-\nulldelimiterspace} {k_{n} }}}} }} $$

with the residual ratio at hyperpolarized voltages (*r*_–∞_) and the maximal ratio (*r*_∞_). The activation time course was measured by applying depolarizing pulses (−20, −10, 0, and 20 mV) of variable lengths (200 ms to 6.5 s) and analyzing the subsequent peak tail currents. For averaging, the data were normalized to the maximal channel activation obtained at 20 mV after 6.5 s.

Tail current deactivation kinetics of Kv10.1 or Kv10.2 channels as a function of the time *t*, starting at the tail segment, were described using a sum of exponential functions according to the binomially distributed occurrence of heteromeric channels. It was assumed that mutant subunits alone (WT: Mut = 0:4, which occur at probability of 1/16) do not produce any measurable current. Homomeric WT channels deactivate with the time constant τ_fast_ (4:0, 1/16). The remaining WT: Mut combinations of 3:1 (4/16), 2:2 (6/16), and 1:3 (4/16), with the binomial factors in parentheses, were described with τ_slow_/*f*^2^ for 3:1, τ_slow_/*f* for 2:2, and τ_slow_ for 1:3, with the scaling factor *f*. *p*_s_, the probability of having a mutant subunit in the open channels at the end of the test pulse, was used as additional fit parameter. The relative amplitudes scale with the probability of the individual combination according to a binomial distribution and a given *p*_s_: *p*(4:0) = (1-*p*_s_)^4^; *p*(3:1) = 4 *p*_s_ (1-*p*_s_)^3^; *p*(2:2) = 6 *p*_s_^2^ (1-*p*_s_)^2^; *p*(1:3) = 4 *p*_s_^3^ (1-*p*_s_); *p*(0:4) = *p*_s_^4^. This model was fit globally to all mean tail currents of a full current-voltage relationship, resulting in the fitted tail current traces and fit parameters shown in Fig. S2.

### Optical recording of the membrane potential

*Life-cell fluorescence imaging.* HEK293T cells were plated in 35-mm glass-bottom dishes (Ibidi, Martinsried, Germany) at an approximate density of 10,000 cells/dish and transfected the following day with plasmids encoding the respective ion channel and mKate2-rEstus [28] (mK2-rEstus) at a 1:1 DNA mass ratio (0.5 µg each) using the ROTI^®^Fect transfection reagent. One day after transfection, cells were washed once with 1 ml external solution composed of (in mM) 146 NaCl, 4 KCl, 2 CaCl_2_, 2 MgCl_2_, and 10 HEPES; pH 7.4 (NaOH). Cells were maintained in 2 ml of the same solution supplemented with 5 mM glucose and 10 µg/ml Hoechst33342 (Invitrogen). Cells were incubated for 30 min at 37 °C and 5% CO_2_ prior to imaging.

Imaging experiments were performed on an Eclipse-Ti fluorescence microscope (Nikon, Tokyo, Japan) equipped with a DS-Qi2 camera (14-bit) and an X-Cite 120 LED light source (Excelitas Technologies, Waltham, MA, USA), operated using NIS Elements 4.6 Software (Nikon). Measurements were performed in an incubation chamber (Oko Lab, Pozzuoli, Italy) maintained at 37 °C. Images from 16 non-overlapping positions per 35-mm dish were acquired using a 10x objective (Plan Apo λ, NA 0.45, Nikon) on an automated stage (H117 stage with ProScan III controller, Prior Scientific, Cambridge, UK). Stage movement and data acquisition were coordinated with NIS-Elements 4.6 software. The z-focus was maintained throughout imaging with the Nikon Perfect Focusing System. At each position, images were acquired in the following channels: four blue excited images for rEstus fluorescence (GFP-3035D-000, 200 ms exposure, camera gain 64), one green-excited image for mKate2 fluorescence (4040 C-000 BrightLine, the filter cube was modified by replacing the original excitation filter with the BP 480/10 filter (Thorlabs), 1 s exposure, camera gain 5.1), and one excitation with UV light for Hoechst33342 fluorescence (DAPI-50-LP-A-000, 1 ms exposure, camera gain 5.1; Semrock filter sets in all cases). In addition, a transmission image was captured.

*Image analysis.* Image analysis was performed using Fiji software [29]. The camera offset for each channel was subtracted, and background of each image was corrected individually using the built-in rolling-ball algorithm (radius 74 μm). Cell nuclei were identified in the blue channel by converting the image into a binary mask with a manually set threshold. The built-in watershed algorithm was used to separate adjacent nuclei that could not be separated by thresholding. Individual nuclei were detected for each image with the particle analysis tool of Fiji. Particles intersecting image borders and those outside of a defined size range (< 7.5 µm^2^ and > 19 µm^2^) were excluded. Regions of interest (ROI) were automatically generated using the particle boundaries defined by the binary mask. Fluorescence parameters were extracted for each individual cell for the green, red, and blue channels. For the green channel, only the fourth image was analyzed to ensure *F*_green_ was determined only after complete photoswitching. Compiled data were further analyzed with Igor Pro (WaveMetrics, Lake Oswego, OR, USA).

Several measures were applied to avoid bias as described previously [30]. In short, cells with very high *F*_red_ signals were excluded from analysis to account for the limited dynamic range of the recording camera. To account for residual autofluorescent medium in the bath, an adaptive background correction was applied. The linear fit of the median *F*_green_/*F*_red_ against *F*_red_ was used to determine the minimum of the squared slope as a function of background *F*_green_. The calculated background *F*_green_ value was subsequently subtracted from *F*_green_ prior to further data analysis. Furthermore, cells with low *F*_red_ intensities were excluded because the error in background subtraction is expected to be largest in low expressing cells. For illustration purposes, pixel-by-pixel *F*_green_/*F*_red_ images were generated in ImageJ. Because adaptive background correction and signal gating were performed on ROI-extracted numerical values in Igor Pro, this processing was not applicable to the ratio images. Therefore, a binary mask derived from the *F*_red_ image was processed with directional ridge-enhancement filters and thresholding and applied to the ratio image to remove background and gated signals. Masked ratio images were displayed in rainbow color coding.

*One-point calibration with gramicidin*. HEK293T cells were treated with 1 µM gramicidin-D (G5002, from a 2 mM stock solution in ethanol) in the extracellular imaging solution for about 30 min until the *F*_green_/*F*_red_ response of the sensor was measured. Gramicidin is an ionophore with weak selectivity among monovalent cations, which ultimately leads to cell depolarization. The medians were measured for each individual cell culture dish. The mean of the individual medians was the considered 0 mV. With this fixed point, all other data were calibrated according to a calibration standard determined under whole-cell voltage-clamp control, as shown previously [28]: the following calibration constants were used: *R* = *F*_green_/*F*_red_, *R*_max_ = 2.86, D*R* = −2.39, *V*_half_ = −31.8 mV, and *k*_s_ = 25.3 mV.

### Chemicals

Chemicals for preparing buffer solutions were purchased from Sigma-Aldrich.

### Data analysis

Electrophysiological data were analyzed using FITMASTER NEXT (Multi Channel Systems MCS) and Igor Pro (WaveMetrics) software. Averaged data are presented as means ± SEM values; individual results are indicated as dots. Groups of data were compared using two-sided unpaired t-test or, as indicated, Wilcoxon rank test, with the resulting *p* values considered as operational data descriptors. The α values of 0.05 were post-hoc adapted when multiple comparisons were applicable.

## Results

### Dominant gain-of-function impact of Kv10.1-G496E on Kv10.1 and Kv10.2 channels

**Fig. 1:**
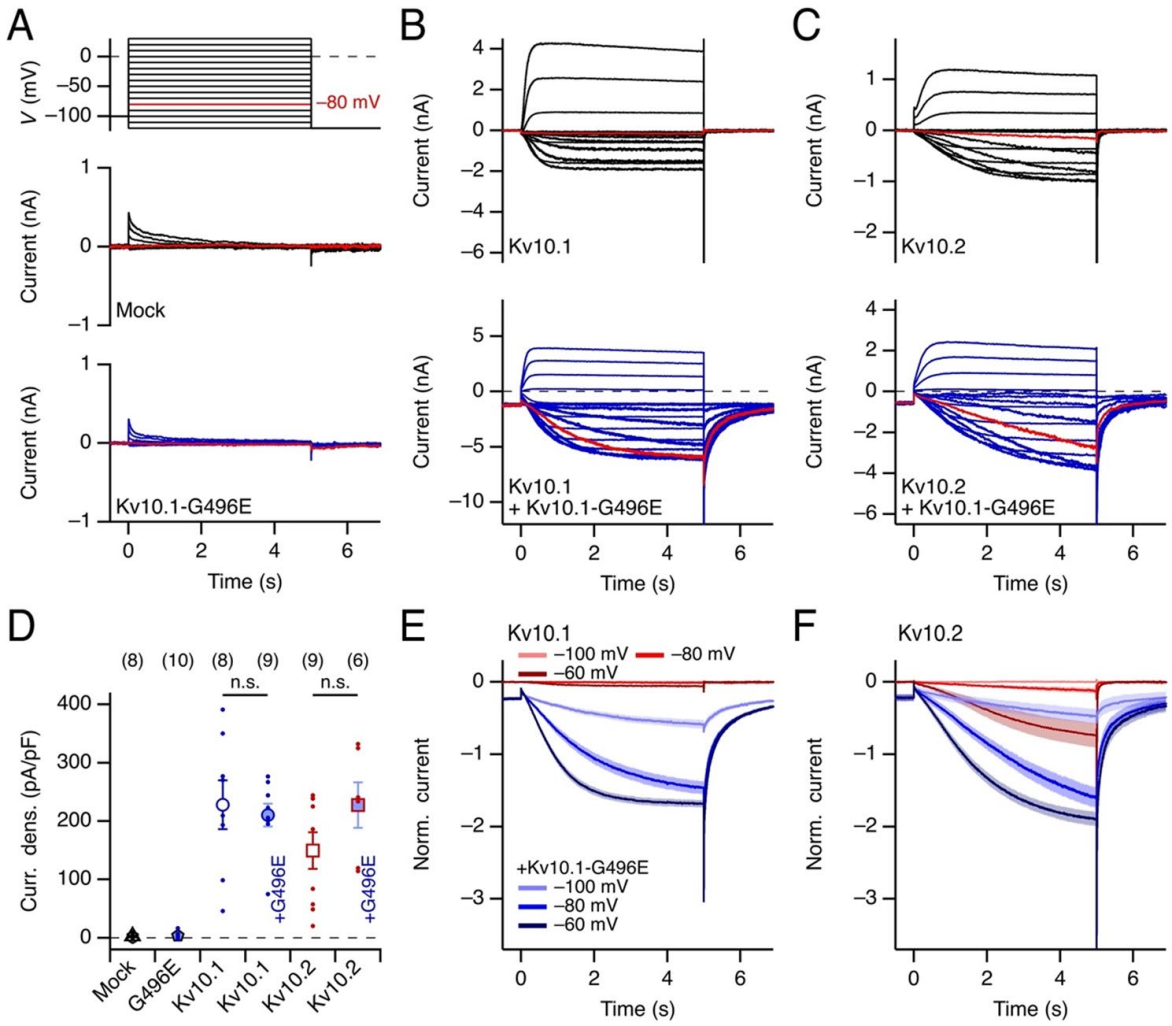
Coexpression of Kv10.1 or Kv10.2 with Kv10.1-G496E. **A** Pulse protocol used to evoke currents from −120 to 30 mV in 10 mV increments (*top*). Superposition of current traces recorded from mock-transfected HEK293T cells (*middle*) and cells expressing Kv10.1-G496E (*bottom*). **B** As in **A**, with expression of Kv10.1 alone (*top*) and Kv10.1 coexpressed with Kv10.1-G496E (*bottom*). **C** As in **B**, for Kv10.2 and Kv10.2 + Kv10.1-G496E. In panels **A**–**C**, the current traces corresponding to a depolarizing pulse to −80 mV are shown in red. **D** Mean current density between 4.9 and 5.0 s at 30 mV from current recordings as shown in **A**–**C**. Error bars represent SEM, with *n* in parentheses; individual data points are displayed as dots; n.s., not significant. **E** Superposition of mean traces, normalized to the steady-state outward current at 30 mV of cells expressing Kv10.1 alone (red) and of cells coexpressing Kv10.1 with Kv10.1-G496E (blue) at the indicated voltages. **F** As in **E**, for Kv10.2 and Kv10.2 + Kv10.1-G496E. Traces in **E** and **F** are mean values with SEM indicated by shading; *n* values are displayed in **D**

To assess the function of K^+^ channels formed by Kv10.1-G496E subunits, the corresponding construct was expressed in HEK293T cells and functionally examined with whole-cell patch clamp. Voltage steps ranging from −120 to 30 mV only evoked minor transient K^+^ currents at high voltage, which were also observed in mock-transfected cells (Fig. 1A and D; *p* = 0.71 testing against mock-transfected cells). Thus, similarly to the previously reported mutation Kv10.1-G496R [12], Kv10.1-G496E alone does not seem to produce functional Kv channels.

Expression of Kv10.1 resulted in voltage-gated K^+^ currents for which channel activation was only noticeable at depolarizations greater than −80 mV (Fig. 1B, *top*). Coexpression of Kv10.1-G496E at about the same ratio produced K^+^ currents that were already activated at resting voltages. As shown in Fig. 1B (*bottom*), even at −120 mV the channels are already partially activated. Superposition of averaged and normalized current traces clearly shows the GoF property that Kv10.1-G496E imposes on wild-type Kv10.1 subunits, with substantial current activation at −60 mV – a voltage at which Kv10.1 homomers are barely active (Fig. 1E). The current density at 30 mV was not affected by the coexpression of Kv10.1-G496E (Fig. 1D; *p* = 0.67). It is also noticeable that channel closure upon repolarization to −120 mV is fast for Kv10.1 monomers (time constant of ~ 8 ms) and becomes much slower in the presence of Kv10.1-G496E (time constant of ~ 5 s). For more detailed tail current analysis, see Fig. S2.

A similar result was obtained when Kv10.1-G496E was coexpressed with Kv10.2 (Fig. 1C and F). As with Kv10.1, Kv10.1-G496E subunits caused the channels to activate at resting voltage and slowed down the tail currents (from 10 ms for the wild type to ~ 5 s with coexpression of Kv10.1-G496E, see Fig. S2). Both results together clearly show that Kv10.1-G496E, which does not produce functional channels as homomers, results in heteromeric channels with Kv10.1 and Kv10.2 subunits that have GoF properties in the sense that channels open at resting membrane voltages.

**Fig. 2:**
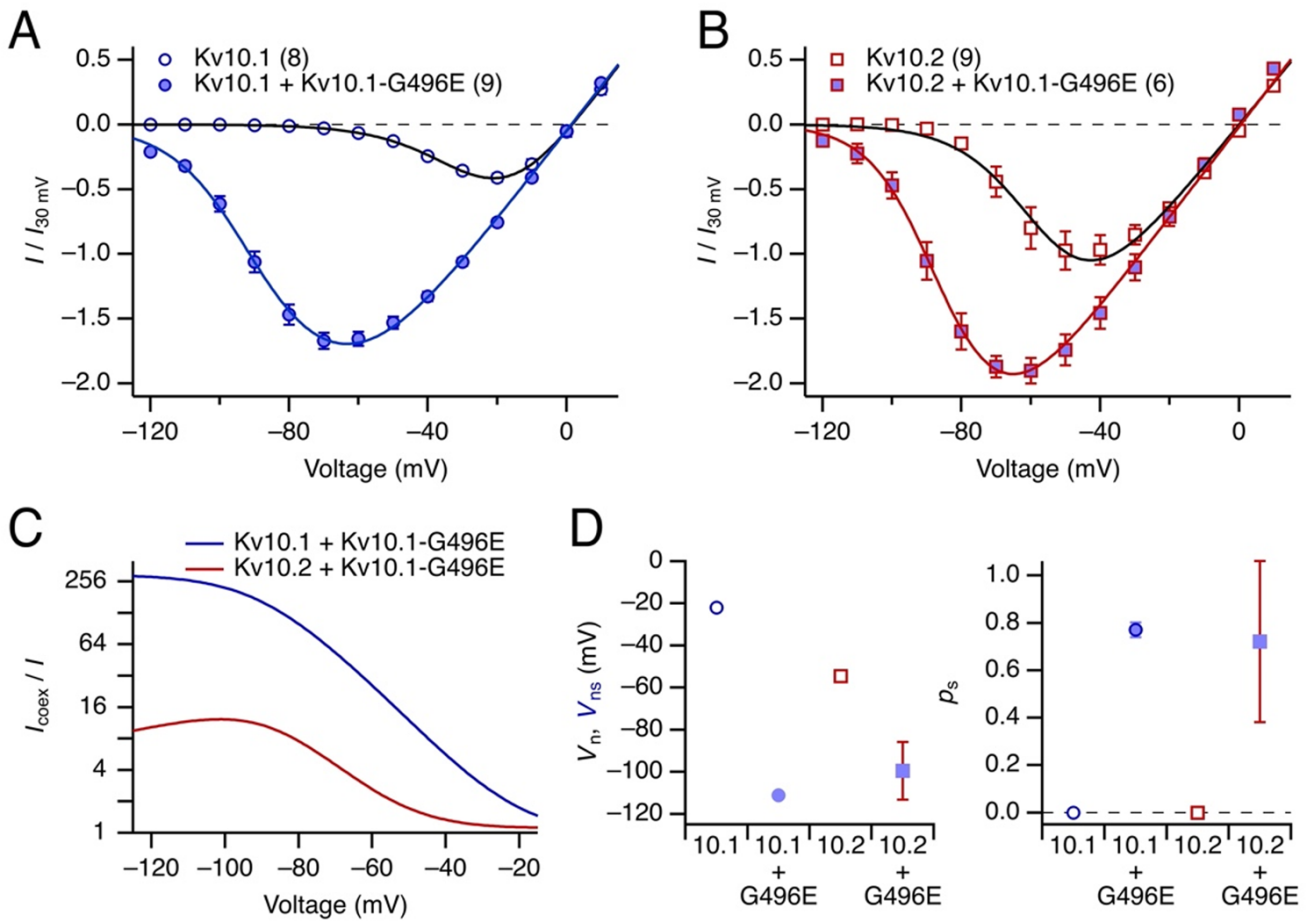
Voltage dependence of channel activation for Kv10.1 or Kv10.2 coexpressed with Kv10.1-G496E. **A** Current-voltage relationships of steady-state current (mean of the last 100 ms of the depolarization) normalized to the current obtained at 30 mV for Kv10.1 (open symbols) and Kv10.1 coexpressed with Kv10.1-G496E (filled blue). The superimposed curves are global data fits to both data sets according to a model for the activation of EAG channels (Eq. (1) and Methods). **B** As in **A**, for Kv10.2 and Kv10.2 together with Kv10.1-G496E. **C** Ratio of current after coexpression of Kv10.1-G496E relative to the expression of Kv10.1 or Kv10.2 alone as a function of voltage. Note that the fractional current increase is displayed on a logarithmic scale. **D** Half-maximal activation voltage (*V*_n_ and *V*_ns_, *left*) and the relative probability of incorporating a mutant subunit into the channel complex (*p*_s_) (*right*) for the indicated channel type without (open symbols) and with coexpression of Kv10.1-G496E (filled blue). Data points are the results of the data fits shown in **A** and **B** with the error bars indicating the 95% confidence intervals

The voltage dependence of channel activation achieved with 5-s depolarizing steps was further analyzed by plotting the current obtained at the end of a 5-s depolarization, normalized to the value for 30 mV, as a function of test voltage (Fig. 2A and B). Even without quantitative analysis it is obvious that Kv10.1-G496E left-shifts the activation voltage with respect to homomeric Kv10.1 (Fig. 2A) and Kv10.2 (Fig. 2B) channels, thus resulting in a substantial GoF at resting voltage. As shown in Fig. 2C, the fractional GoF is voltage dependent and in magnitude greater for Kv10.1 than for Kv10.2 channels.

For quantification, we fit the voltage dependence of the homomeric wild-type currents assuming channel activation, characterized by *V*_n_ and *k*_n_ (Eq. 1). For the currents resulting from coexpression of Kv10.1-G496E we assumed that homomeric mutant channels are not functional. We further assumed that the gating of the mutant subunits is characterized by *V*_ns_ and *k*_ns_, with the index “s” referring to slow activation/deactivation. To minimize the number of free parameters, *k*_ns_ was held at the value of *k*_n_. Furthermore, we assumed that the average fraction of mutant subunits in all channels is *p*_s_. With this assumption, the current-voltage relationships were fit, resulting in the parameters shown in Fig. 2D. Remarkably, this analysis predicts *V*_ns_ values for Kv10.1-G496E subunits of about −100 mV (−111 ± 1.5 mV from the coexpression with Kv10.1 and −99.5 ± 13.7 mV from the coexpression with Kv10.2) and a *p*_s_ of about 0.6-0.8. The latter implies that Kv10.1-G496E coassembles about as well with Kv10.2 as it does with Kv10.1. This analysis also explains why the GoF impact of Kv10.1-G496E is apparently weaker on Kv10.2 than on Kv10.1: *V*_n_ of Kv10.2 (−54.4 ± 1.2 mV) is already strongly left-shifted with respect to Kv10.1 (−22.0 ± 0.4 mV).

For a quantitative description of the deactivation kinetics at −120 mV we assumed a binomial distribution of kinetic components according to the subunit composition of the channels: homomeric mutant channels are not functional and wild-type channels deactivate with the time constant τ_fast_; channels formed of three Kv10.1-G496E subunits and one wild-type subunit deactivate with time constant τ_slow_; channels formed by two wild-type and two mutant subunits deactivate with τ_slow_/*f*, and a 3:1 ratio results in a deactivation time constant of τ_slow_/*f*^2^. As shown in Fig. S2, this simple assumption results in adequate descriptions of the deactivation kinetics with a τ_slow_ value of 5 s and a factor *f* = 5. The results furthermore reflect how the contribution of wild-type subunits becomes more prominent with increasing depolarization, as active channels formed by mostly wild-type subunits are underrepresented at weak depolarizations (Fig. 2).

Qualitatively similar results were obtained for the coexpression of Kv10.1-G496E with Kv10.2. Also in this case, fast tail currents (time constant at −120 mV of about 10 ms) became much slower when Kv10.1-G496E was coexpressed, indicating that the tail current kinetics are determined by the number of mutant subunits incorporated into a functional channel complex (Fig. S2).

It should be noted that further refinement of the kinetic model is hampered by the following technical shortcomings: Owing to channel activation at very negative voltages, no p/n leak correction can be performed. In particular for Kv10.2 channels, the activation kinetics are too slow to reach a steady-state at all voltages with depolarizing pulses of 5 s. Furthermore, the exact subunit ratio in HEK293T cells cannot be fully controlled in coexpression experiments.

The mutation G496E in the long splice form (Kv10.1_long_) shows similar properties as Kv10.1-G496E: Kv10.1_long_-G496E does not produce functional K^+^ channels when expressed alone and this mutation has a GoF impact on Kv10.2 when coexpressed (Fig. S3).

### Reverse mutagenesis of Kv10.2

The results shown thus far demonstrate how a disease-causing mutation of Kv10.1 subunits (Kv10.1-G496E) can exert a GoF impact on Kv10.1 and Kv10.2 channels. The reverse scenario was tested by creating and evaluating an equivalent mutation in Kv10.2 at the conserved glycine residue 465: Kv10.2-G465E. As shown in Figs. S4 and S5, this mutant alone does not result in functional channels, and coexpression with Kv10.1 or Kv10.2 imposes a GoF effect on these channels by left-shifting the voltage dependence of activation. Thus, GoF mutants of *KCNH5* are also expected to alter the function of Kv10.1 channels when expressed in the same cells.

### Lack of obvious functional crosstalk with Kv11.1

**Fig. 3:**
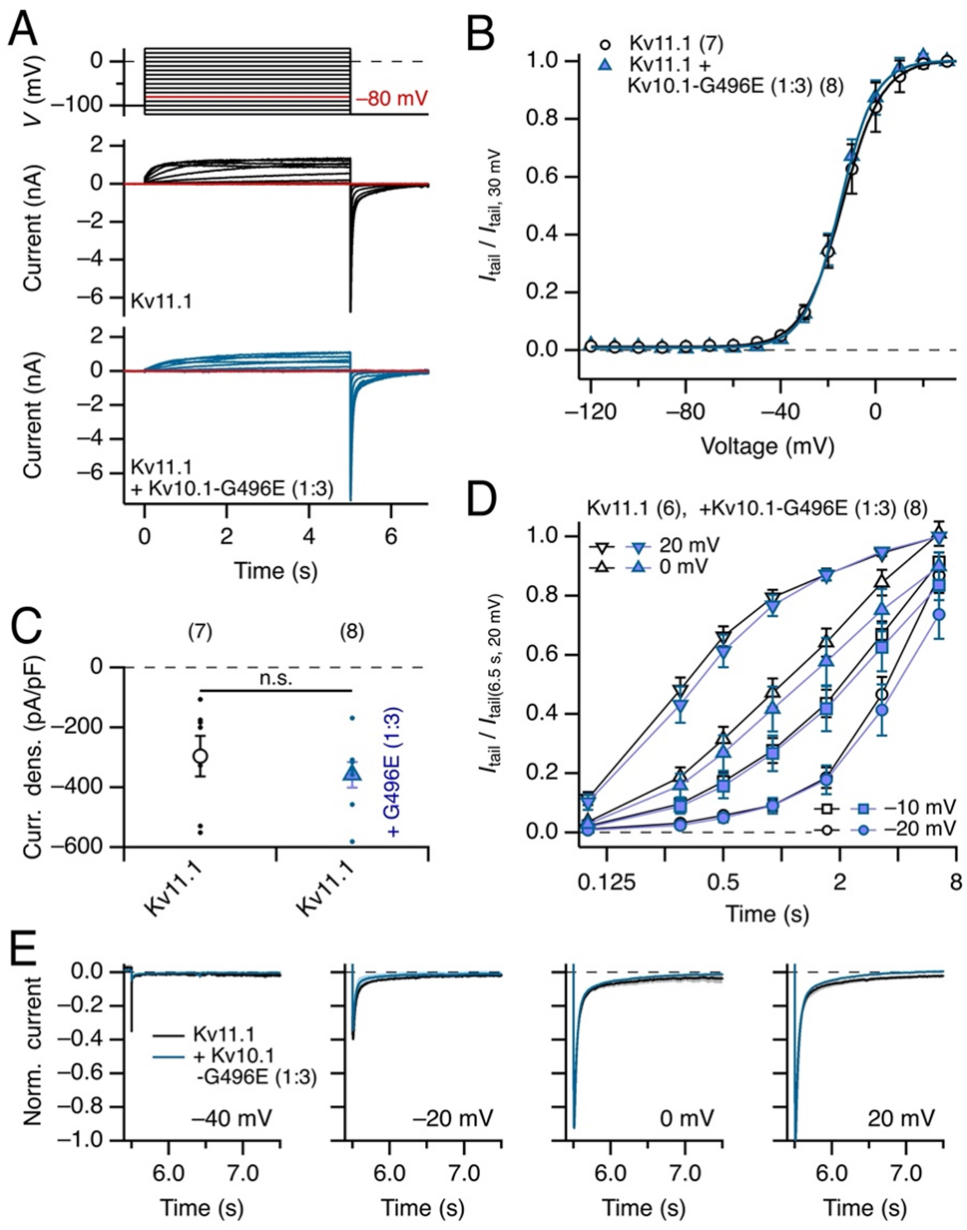
Coexpression of Kv11.1 with Kv10.1-G465E. **A** Pulse protocol used to evoke currents from −120 to 30 mV in 10 mV increments (*top*). Superposition of current traces recorded from HEK293T cells expressing Kv11.1 (*middle*) and cells coexpressing Kv11.1 with Kv10.1-G496E at an approximate DNA weight ratio of 1:3 (*bottom*). **B** Tail current-voltage relationships normalized to the current obtained after depolarization to 30 mV for Kv11.1 (black) and the indicated coexpression with Kv10.1-G496E (petrol). The superimposed curves are fit functions according to Eq. (2). **C** Mean tail-current density at −120 mV after depolarization to 30 mV from current recordings as shown in **A**. Error bars represent SEM, with *n* in parentheses; n.s., not significant; individual data points are displayed as dots. **D** Mean tail currents at −120 mV after depolarization to the indicated voltages as a function of depolarization duration. Data points are means ± SEM, *n* in parentheses. There are no significant differences between Kv11.1 (open symbols) and its coexpression with Kv10.1-G496E (filled symbols) for all conditions (*p* = 0.11-0.99). Straight lines connect the data points for clarity. **E** Superimposed mean tail currents at −120 mV for Kv11.1 (black) and Kv11.1 + Kv10.1-G496E (petrol) for the indicated test potentials. The dark lines are means (for *n*, see **B**, SEM is indicated by shading)

As closest relatives of Kv10.1 and Kv10.2 channels, we also examined a potential impact of Kv10.1-G496E coexpression with Kv11.1 (hERG1) subunits. In a 1:1 ratio of DNA plasmids used for coexpression, we did not find any indication that current magnitude, voltage dependence, or kinetics of Kv11.1 channels were affected by the presence of Kv10.1-G496E (not shown). Even at a wild type to mutant DNA ratio of 1:3, no indication of functional alteration was observed (Fig. 3): the voltage dependence of tail current activation (Fig. 3B), the current density (Fig. 3C), the kinetics of channel activation (Fig. 3D), and the kinetics of channel deactivation at −120 mV (Fig. 3E) were not altered by coexpression of Kv10.1-G496E.

### Impact of *KCNH1/5* expression on the membrane potential

**Fig. 4:**
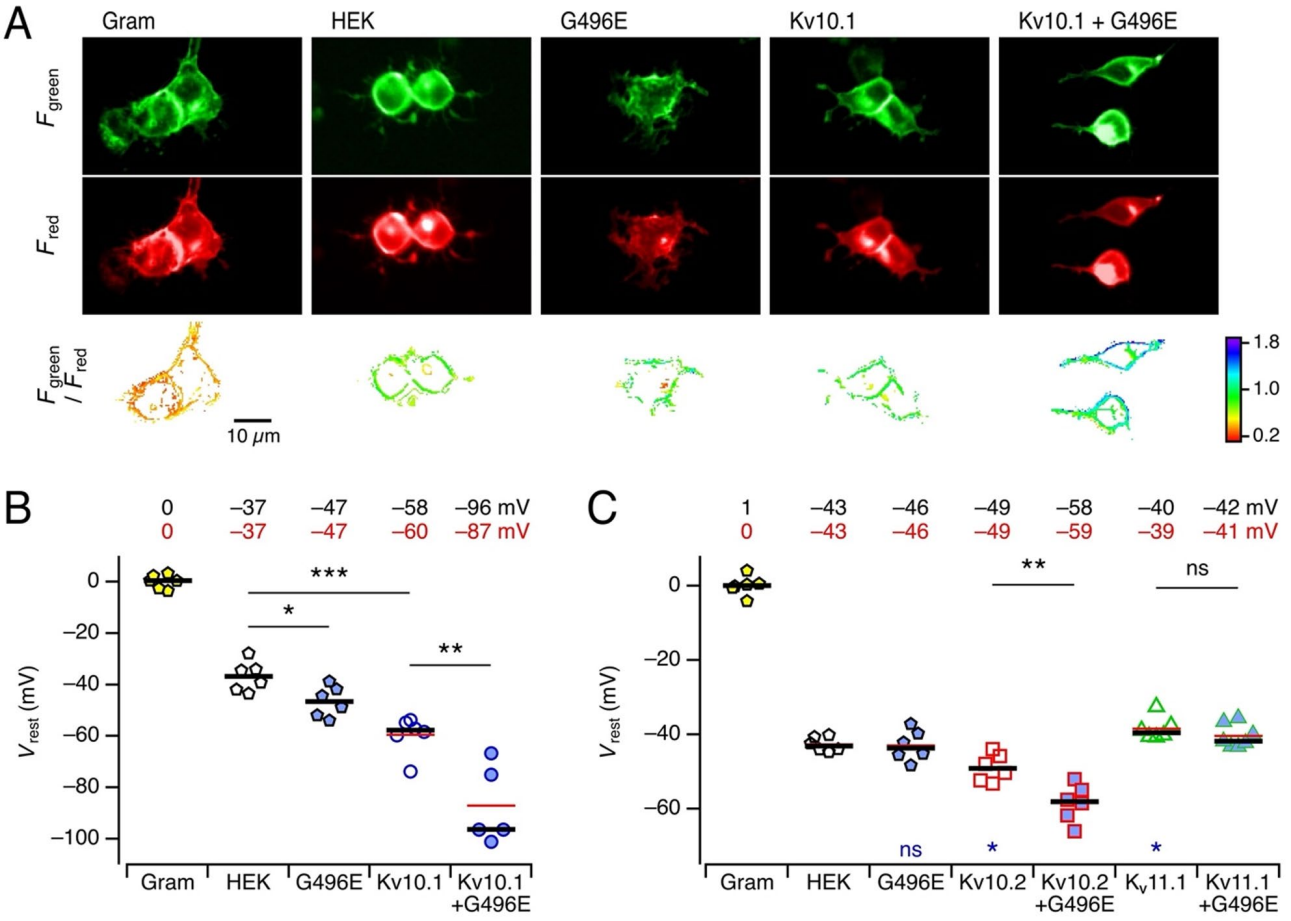
Assessment of the resting membrane voltage of HEK293T cells with a genetically encoded voltage indicator. **A** Fluorescence images of HEK293T cells expressing mK2-rEstus with the indicated expression constructs and treatments. Gram = gramicidin, HEK = HEK293T without channel expression, G496E = Kv10.1-G496E. *top*, Voltage-sensitive green fluorescence; *middle*, voltage-insensitive mKate2 fluorescence; *bottom*, ratio image after edge detection. **B** Median resting voltages of HEK293T cells with the indicated expression constructs and treatments; K_V_10.1-G496E coexpression is indicated by blue fill color. Black horizontal bars indicate the medians – also given numerically at the top; the red values and horizontal red lines indicate the means. Each data point is the result of cells from one culture dish, *n* = 1290 ± 160 cells (min 259, max 3411). **C** As in **B**, with independent gramicidin calibration and coexpression of Kv10.1-G496E with Kv10.2 and K_V_11.1. Each data point is the result of cells from one culture dish, *n* = 2650 ± 140 cells (min 925, max 4845). Wilcoxon rank test: ***p < 0.001, **p < 0.01, *p < 0.05, ns not significant. The blue symbols in **C** are results of a Wilcoxon rank test versus HEK293T cells with post-hoc correction for multiple comparisons. For the distributions of all cells, see Fig. S6

The results thus far shown are based on whole-cell patch-clamp recordings with high extracellular K^+^ concentration to visualize channel activation at resting voltages and with intracellular solutions devoid of Na^+^ and Ca^2+^ ions. While these experimental conditions are suitable for characterizing the K^+^ channel function, they do not permit a direct conclusion about the function under physiological conditions, particularly when considering the dependence of the Kv10 channels on the intracellular Ca^2+^ concentration [22] and the PIP_2_ level [23]. To approximate a physiological scenario, we employed a fluorescence-based assay to record the resting membrane voltage (*V*_rest_) in a noninvasive manner. The assay involved mK2-rEstus, which comprises the green-fluorescent genetically encoded voltage indicator rEstus [28] with an N-terminally fused red fluorescence protein mKate2 [30]. While the green fluorescence (*F*_green_) of rEstus depends on the membrane voltage, the red fluorescence (*F*_red_) of mKate2 is independent of voltage and serves for calibration. An increase in *F*_green_/*F*_red_ reports cell hyperpolarization (Fig. 4A).

For the final experiments with transfected HEK293T cells, the true *V*_rest_ was obtained from a prior calibration (Methods) and a measurement at 0 mV, achieved with gramicidin-mediated cell depolarization. Figure 4B shows one of these experiments in which for each condition six cell culture dishes (each with several hundred cells) were examined and the measured *F*_green_/*F*_red_ converted to membrane voltages (for the distributions of all cells, see Fig. S6). While the mean *V*_rest_ of resting HEK293T cells was about −37 mV, expression of Kv10.1-G496E slightly hyperpolarized the cells to −47 mV, and Kv10.1 significantly hyperpolarized the cells to −60 mV. Coexpression of Kv10.1 with Kv10.1-G496E significantly hyperpolarized the cells further to −87 mV. In a second independent set of experiments (Fig. 4C), HEK293T cells of this batch were slightly more negative (−43 mV), and expression of Kv10.1-G496E did not significantly hyperpolarize the cells further. Kv10.2 caused a weak hyperpolarization to −49 mV, while coexpression of Kv10.2 with Kv10.1-G496E hyperpolarized further to −59 mV. The degree of hyperpolarization was less than that obtained with the coexpression of Kv10.1 and Kv10.1-G496E, which is consistent with the current properties reported in Figs. 1 and 2. Finally, Kv10.1-G496E did not change *V*_rest_ of cells that also expressed Kv11.1. Thus, this noninvasive assay recapitulates the results obtained from whole-cell recordings, namely that Kv10.1-G496E had no obvious impact on the cells when expressed alone. Both, Kv10.1 and Kv10.2 have the propensity to hyperpolarize HEK293T cells, and in the background of either Kv10.1 or Kv10.2 the mutant Kv10.1-G496E imposes a strong GoF effect, here visualized as additional cell hyperpolarization. Such hyperpolarization is not observed when Kv10.1-G496E is coexpressed with Kv11.1 subunits.

## Discussion

Our study demonstrates that GoF mutations in *KCNH1* and *KCNH5* are not restricted to their respective homomeric channel complexes but can profoundly influence each other’s function through heteromeric assembly. The Kv10.1-G496E and Kv10.2-G465E mutations, although incapable of producing functional channels when expressed alone, caused strong hyperpolarizing shifts in the voltage dependence of activation in both Kv10.1- and Kv10.2-based channels when coexpressed. These findings provide direct evidence that pathogenic mutations in one *KCNH* gene can reshape the functional properties of channels formed by the paralogous gene, thereby introducing an additional layer of complexity to genotype–phenotype correlations in Kv10-related developmental disorders.

Attempts to relate clinical presentations to specific *KCNH1* or *KCNH5* mutations have been complicated by the wide phenotypic spectra of ZLS, TBS, and the emerging *KCNH5*-associated epileptic encephalopathies [14, 31]. Most analyses implicitly treat *KCNH1*- and *KCNH5*-mediated disorders as independent entities. Our data argue that the molecular pathophysiology of Kv10 channelopathies must consider the potential impact of heteromeric channels. The large left-shifts observed in coexpressed channels indicate that a single mutant subunit can dominate the gating behavior of the entire tetramer. Importantly, the fractional incorporation estimates indicate efficient coassembly of Kv10.1-G496E with both Kv10.1 and Kv10.2, consistent with the known plasticity of subfamily-intrinsic heterotetramerization.

A prerequisite for heteromer formation is that *KCNH1* and *KCNH5* must be expressed in overlapping anatomical regions and even within the same neuronal populations. Early in situ hybridization-based studies of expression patterns in rat brain found that *Kcnh1* (rEag1) and *Kcnh5* (rEag2) have overlapping expression in the cerebral cortex, with high expression of both genes in, e.g., Layers III and IV [24, 32]. By contrast, more complementary expression patterns were found for other areas, such as thalamus and pons, with predominant expression of *Kcnh5* or the cerebellum with exclusively high expression of *Kcnh1*. Later studies based on RNA sequencing largely confirmed this anatomical expression landscape also for the human brain. Data available in The Human Protein Atlas (www.proteinatlas.org/ENSG00000143473-KCNH1/brain and /ENSG00000140015-KCNH5/brain; accessed Dec 2025) show high expression of both paralogs in cerebral cortex, high levels of *KCNH5* in thalamus and pons, and high *KCNH1* expression in the cerebellum. Importantly, evidence for coexpression of both genes extends to the single-cell level. Analysis of human cortical single-nucleus RNA-seq datasets available through the CZ CELLxGENE portal [33] indicates that *KCNH1* and *KCNH5* are broadly expressed in various glutamatergic neuron subclasses. Notably, in Layer-III intratelencephalic (IT) projecting glutamatergic neurons, approximately 70% of cells express *KCNH1* and 54% express *KCNH5*, while in Layer-IV IT neurons, 62% and 37% of cells express *KCNH1* and *KCNH5*, respectively. The coexpression of *KCNH1* and *KCNH5* in glutamatergic neurons of cortical Layers III and IV is particularly noteworthy, as excitatory microcircuits in these layers might be involved in the initiation and propagation of epileptic discharges [34, 35].

We found no evidence that Kv10.1-G496E interacts functionally with Kv11.1, despite its close evolutionary relationship. This specificity is consistent with previous studies showing that heteromerization within *KCNH* subfamilies is permitted, whereas inter-subfamily combinations are excluded [8, 10, 11]. Together, these observations reinforce the idea that pathogenic interactions are likely confined to cells coexpressing *KCNH1* and *KCNH5*.

Our findings have important implications for the clinical interpretation of *KCNH* variants. The presence of a pathogenic mutation in either *KCNH1* or *KCNH5* may alter the functional landscape of both channels, complicating direct genotype–phenotype mapping. Variant classification should therefore consider not only the functional effect of a mutation in isolation but also the potential coexpression context in relevant tissues. Patients carrying variants in one gene might exhibit symptoms traditionally associated with the other gene. This concept may help explain the clinical overlap between TBS, ZLS, and *KCNH5*-related epileptic encephalopathy, as well as the absence of defining dysmorphic features, such as gingival fibromatosis or nail aplasia in *KCNH5*-related cases.

All experiments were performed in HEK293T cells, which provide a system for studying subunit interactions but cannot fully replicate neuronal conditions. The relative stoichiometry of subunits in native neurons may differ from our expression conditions, and future studies using induced pluripotent stem cell-derived neurons or mouse models will be essential to determine how physiological expression patterns shape the influence of mutant subunits. Moreover, it will be important to examine additional disease-associated mutations to assess whether dominant GoF through heteromerization is a generalizable mechanism in the Kv10 subfamily.

Mutations in *KCNH1* causing GoF properties are found in various parts of the protein, but several mutations (e.g [12, 13, 26]) are clustered at the end of the transmembrane segment 6 (Fig. S1A [36]). In this area, residue G496 is highly conserved in various species [26] and between human Kv10.1 and Kv10.2 (Fig. S1B). The alteration G496R has been described by Kortüm *et al.* [12], who found that this variant by itself does not form functional K^+^ channels but imposes GoF properties on Kv10.1 wild-type subunits. Here, we report the functional properties of G496E [26]: although the alteration G496E introduces the opposite charge compared to the previously described G496R, the functional impact is similar. This indicates that the general disturbance of the structure at G496, e.g., as to make it bulkier or less hydrophobic, impacts the closing of the channel, possibly by unmasking a normally hidden conducting state [37]. It should be noted that G496 in domain-swapping Kv channels is a gating hinge, while in non-domain-swapping Kv11.1 channels of the *KCNH* family, it does not seem to serve the same purpose [38].

Despite the technical limitations that compromise the establishment of a detailed gating scheme, the model-dependent analysis of the voltage dependence of channel opening and its deactivation kinetics (Figs. 2 and S2) is compatible with the notion that Kv10.1 and Kv10.2 channels tolerate up to three G496E subunits (Kv10.1-G496E or Kv10.2-G465E), and even one mutated subunit in the channel complex induces a GoF phenotype by shifting the voltage of activation in the direction of the resting potential and by slowing channel deactivation. As this functional crosstalk between Kv10.1 and Kv10.2 is bidirectional, the impact caused by the mutations Kv10.1-G496E and Kv10.2-G465E appears weaker for Kv10.2 channels because these channels open at lower voltages than Kv10.1 (Fig. 2D). Regarding the likelihood of coassembly of channel-forming subunits, we found neither a preference between Kv10.1 and Kv10.2 nor between wild-type and mutated subunits. Thus, these subunits appear to form channel complexes without significant structural constraints.

For the assessment of the channel properties, we conducted whole-cell patch-clamp experiments with symmetrical K^+^ buffers to visualize channel opening at resting voltages. Furthermore, the intracellular solution contained 10 mM EGTA to chelate Ca^2+^ ions. While the biophysical analysis clearly shows that alteration Kv10.1-G496E induces a GoF phenotype in Kv10.1 and also in Kv10.2 channels, it remains elusive which cell functions are affected by the expression of mutated Kv10.1 or Kv10.2, and what might be the final physiological or pathophysiological outcome. Since the resting *V*_rest_ is not only an important parameter for the electrical excitability of neurons but also emerges as a key factor in development (e.g [39, 40]), we investigated the impact of wild-type and mutant subunits on *V*_rest_ of HEK293T cells using a noninvasive optical assay. The two-color genetically encoded voltage indicator mK2-rEstus revealed the propensity of Kv10.1 and Kv10.2 channels to slightly hyperpolarize the cells, while the coexpression of Kv10.1-G496E substantially enhanced this effect. The assay is therefore useful for the noninvasive and rapid evaluation of *KCNH1* or *KCNH5* GoF mutations, and it shows that a GoF phenotype is not counteracted by compensatory mechanisms such as Ca^2+^/calmodulin-mediated channel inhibition.

In summary, we identify heteromeric crosstalk between Kv10.1 and Kv10.2 as a key mechanism by which gain-of-function mutations may propagate their effects, extending pathogenic influence beyond the mutated gene itself. This mechanism provides a possible explanation for the variability of neurological phenotypes associated with *KCNH1* and *KCNH5* mutations and highlights the importance of considering subfamily-wide interactions in understanding channelopathies.

## Supplementary Information

Below is the link to the electronic supplementary material.

Supplementary Material 1

## Data Availability

All data generated or analyzed during this study are included in this published article and its supplementary information.

## Abbreviations

ASD: Autism spectrum disorders
EAG: Ether à go-go
ELK: EAG-like K^+^ channel
ERG: EAG-related gene
GoF: Gain of function
IT: Intratelencephalic
Kv: Voltage-gated K^+^
ROI: Region of interest
TBS: Temple-Baraitser syndrome
*V*_rest_: Resting membrane voltage
ZLS: Zimmermann-Laband syndrome
