# Supplementary material for: Crosstalk of KCNH1 and KCNH5 gain-of-function mutations leading to epilepsy and neurodevelopmental disorders
Source: Mol Brain. 2026 Feb 8;19:16. doi: 10.1186/s13041-026-01279-1 (PMC12931077; doi:10.1186/s13041-026-01279-1)
Supplement: Supplementary file 1 — Supplementary Material 1 [file 13041_2026_1279_MOESM1_ESM.pdf]

Crosstalk of *KCNH1* and *KCNH5* gain-of-function mutations leading to epilepsy and neurodevelopmental disorders

Supplementary Information

Alisa Bernert[a], Philipp Rühl[a], Roland Schönherr[a] and Stefan H. Heinemann[a,*]

[a] *Center for Molecular Biomedicine, Department of Biophysics,*
Friedrich Schiller University Jena and Jena University Hospital, Jena, Germany

* Corresponding author
Center for Molecular Biomedicine, Department of Biophysics,
Friedrich Schiller University Jena and Jena University Hospital,
Hans-Knöll-Straße 2, 07745 Jena, Germany
stefan.h.heinemann@uni-jena.de
ORCID: 0000-0002-4144-0251

Supplementary Figures

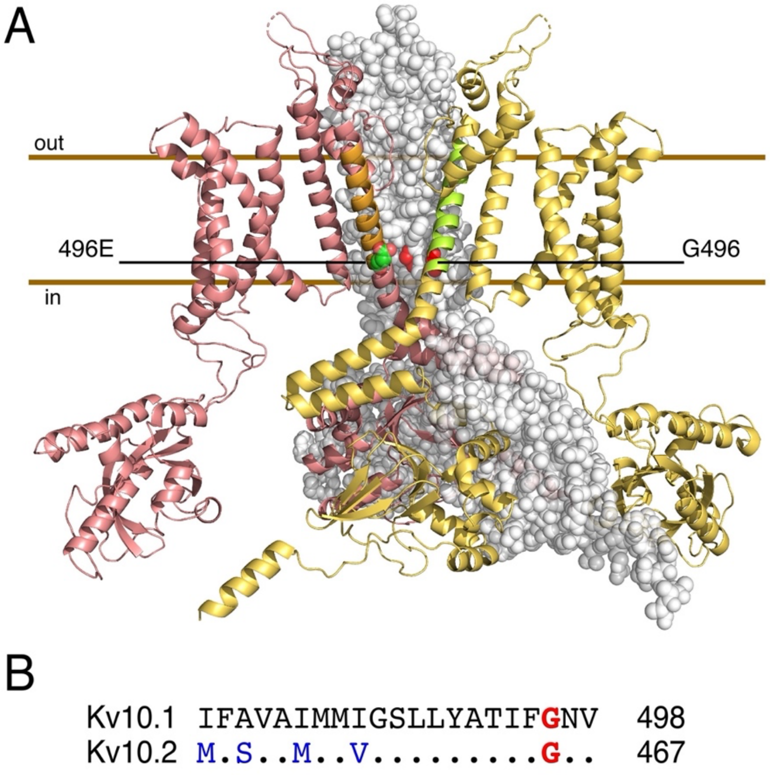

Supplementary Figure 1 Location and conservation of Kv10.1-G496. **A**) Partial cryo-EM structure of rat EAG1 (Kv10.1) (from PDB 5K7L (1)) without calmodulin and only showing three of four subunits; one subunit in the back shown as spheres. Transmembrane segment 6 (S6) according to UniProt is highlighted in orange (left) and limon (right). Residue G496 is shown as red spheres in two subunits; in the subunit shown in salmon, a glutamate residue was inserted instead of G496. The image was generated using PyMOL 2.5.2. **B**) Alignment of the human Kv10.1 and Kv10.2 protein sequences (according to UniProt) encompassing S6. Dots indicate conservation, nonconserved residues are shown in blue, and G496 (Kv10.1) and G465 (Kv10.2) in red. The number of the last residue is indicated (in Kv10.1 for the long “b” splice variant).

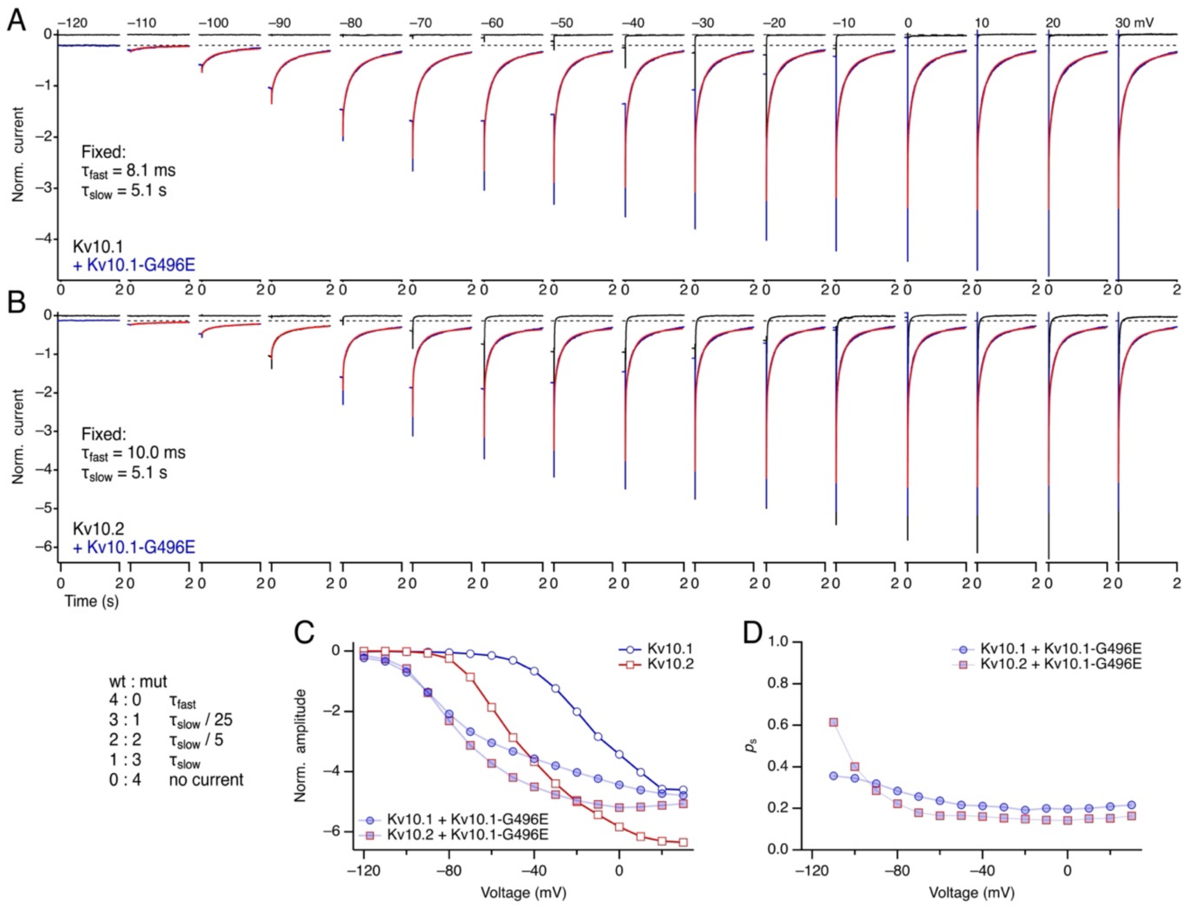

Supplementary Figure 2 Tail current kinetics of Kv10.1 or Kv10.2 coexpressed with Kv10.1-G496E. Mean normalized current traces, as in Fig. 1F and G, in a presentation showing the deactivation phase at -120 mV only. The black traces refer to the control expression of Kv10.1 (**A**) and Kv10.2 (**B**); the blue traces are from cells in which Kv10.1-G496E was coexpressed. The superimposed red curves are global data fits to the entire data sets assuming that channels formed of four Kv10.1 or Kv10.2 subunits show rapid deactivation (τ_{fast}), and channels formed of four Kv10.1-G496E subunits do not produce any current. It is further assumed that channels with three Kv10.1-G496E subunits exhibit slow deactivation (τ_{slow}). Fewer Kv10.1-G496E subunits diminish the deactivation speed: for two Kv10.1-G496E subunits τ becomes τ_{slow}/f; for one Kv10.1-G496E subunits it becomes τ_{slow}/f^2. This approach yields the following global parameters: τ_{fast}, τ_{slow}, and $f = 5$; it furthermore yields as individual voltage-dependent parameters the maximal amplitude and the average ratio of Kv10.1-G496E over control subunits incorporated in channels that are activated at the end of the test pulse (p_s). **C**) Maximal tail current amplitude as a function of the test-pulse voltage. **D**) p_s, based on the fits in A) and B), as a function of the test-pulse voltage. Straight lines connect data points in C) and D) for clarity.

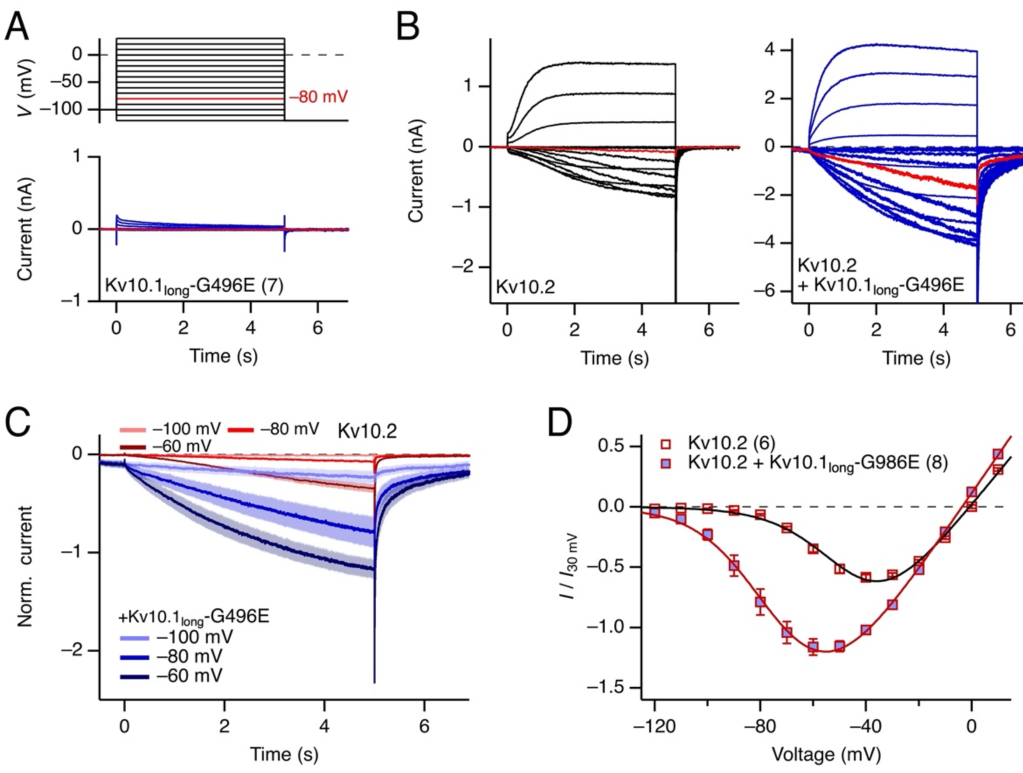

Supplementary Figure 3 Coexpression of Kv10.1$_{long}$-G496E with Kv10.2. **A**) Pulse protocol used to evoke currents from -120 to 30 mV in 10 mV increments (*top*) and superposition of mean currents of HEK293T cells expressing Kv10.1$_{long}$-G496E (*bottom*). **B**) Superposition of current traces from representative experiments from cells expressing Kv10.2 alone (*left*) and Kv10.2 together with Kv10.1$_{long}$-G496E (*right*). The current trace with depolarization to -80 mV is shown in red.
C) Superposition of mean traces at the indicated voltages, normalized to the steady-state outward current at 30 mV, of cells expressing Kv10.2 alone (red) and of cells expressing Kv10.2 together with Kv10.1$_{long}$-G496E (blue). For *n* values, see D). **D**) Current-voltage relationships of steady-state current (mean of the last 100 ms of the depolarization) normalized to the current obtained at 30 mV for Kv10.2 (open symbols) and Kv10.2 coexpressed with Kv10.1$_{long}$-G496E (filled blue). Superimposed fit curves are data fits as in Fig. 2 resulting in the following fit parameters: Kv10.2: V_n = 43.9 ± 1.6 mV, k_n = 11.9 ± 0.7 mV; Kv10.2 coexpressed with Kv10.1$_{long}$-G496E: V_{ns}, = -91.4 ± 3.4 mV, p_s = 0.59 ± 0.07. These results indicate that Kv10.2 and Kv10.1$_{long}$-G496E form functional heteromeric channels with gain-of-function properties compared to Kv10.2 alone.

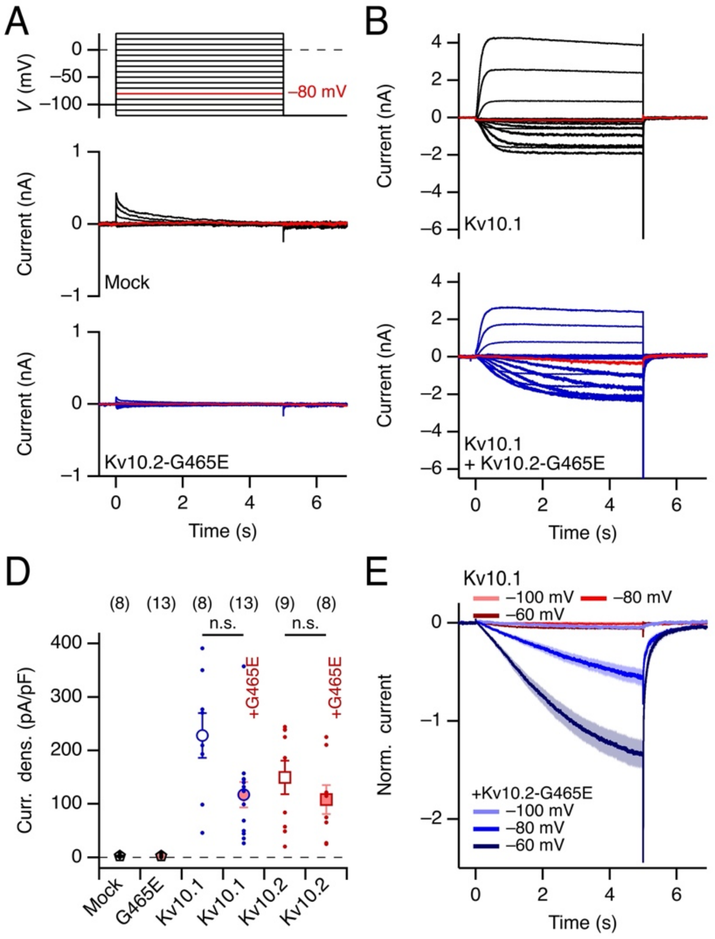

Supplementary Figure 4 Coexpression of Kv10.1 or Kv10.2 with Kv10.2-G465E. **A**) Pulse protocol used to evoke currents from -120 to 30 mV in 10 mV increments (*top*). Superposition of current traces recorded from mock-transfected HEK293T cells (*middle*) and cells expressing Kv10.2-G465E (*bottom*). **B**) As in A), with expression of Kv10.1 alone (*top*) and Kv10.1 coexpressed with Kv10.2-G465E (*bottom*). **C**) As in B), for Kv10.2 and Kv10.2 + Kv10.2-G465E. In panels A)-C), the current traces corresponding to a depolarizing pulse to -80 mV are shown in red. **D**) Mean current density between 4.9 and 5.0 s at 30 mV from current recordings as shown in A)-C). Error bars represent SEM, with *n* in parentheses; individual data points are displayed as dots; n.s., not significant. **E**) Superposition of mean traces, normalized to the steady-state outward current at 30 mV of cells expressing Kv10.1 alone (red) and of cells coexpressing Kv10.1 with Kv10.2-G465E (blue) at the indicated voltages. **F**) As in E), for Kv10.2 and Kv10.2 + Kv10.2-G465E. Traces in E) and F) are mean values with SEM indicated by shading; *n* values are displayed in D). Mock, Kv10.1, and Kv10.2 control data are identical to the data shown in Fig. 1.

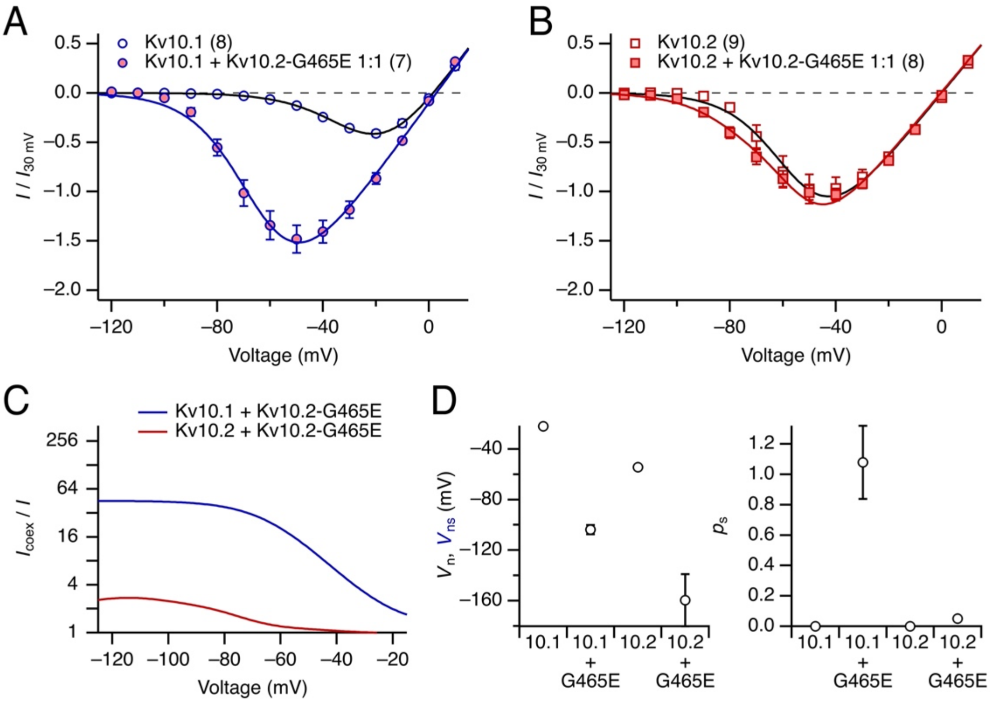

Supplementary Figure 5 Voltage dependence of channel activation for Kv10.1 or Kv10.2 coexpressed with Kv10.2-G465E. **A**) Current-voltage relationships of steady-state current (mean of the last 100 ms of the depolarization) normalized to the current obtained at 30 mV for Kv10.1 (open symbols) and Kv10.1 coexpressed with Kv10.2-G465E (filled pink). The superimposed curves are global data fits to both data sets according to a model for the activation of EAG channels (Eq. (1) and Methods). **B**) As in A), for Kv10.2 and Kv10.2 together with Kv10.2-G465E. **C**) Ratio of current after coexpression of Kv10.2-G465E relative to the expression of Kv10.1 or Kv10.2 alone as a function of voltage. Note that the fractional current increase is displayed on a logarithmic scale. **D**) Half-maximal activation voltage (V_n and V_{ns}, *left*) and the relative probability of incorporating a mutant subunit into the channel complex (p_s) (*right*) for the indicated channel type without (open symbols) and with coexpression of Kv10.2-G465E (filled pink). Data points are the results of the data fits shown in A) and B) with the error bars indicating the 95% confidence intervals. Kv10.1 and Kv10.2 control data are identical to Fig. 2. The model did not fit the data well, as becomes obvious from the resulting p_s values, which were unreasonably large for Kv10.1+Kv10.2-G465E and small for Kv10.2+Kv10.2-G465E. Besides the experimental limitations for estimating true steady-state channel activation, it is possible that the slope factor for the Kv10.2-G465E subunits (k_{hs}) significantly deviates from that of the wild type. The result in C) should therefore considered as a mode-independent estimate of the GoF effect imposed by the coexpression of Kv10.2-G465E.

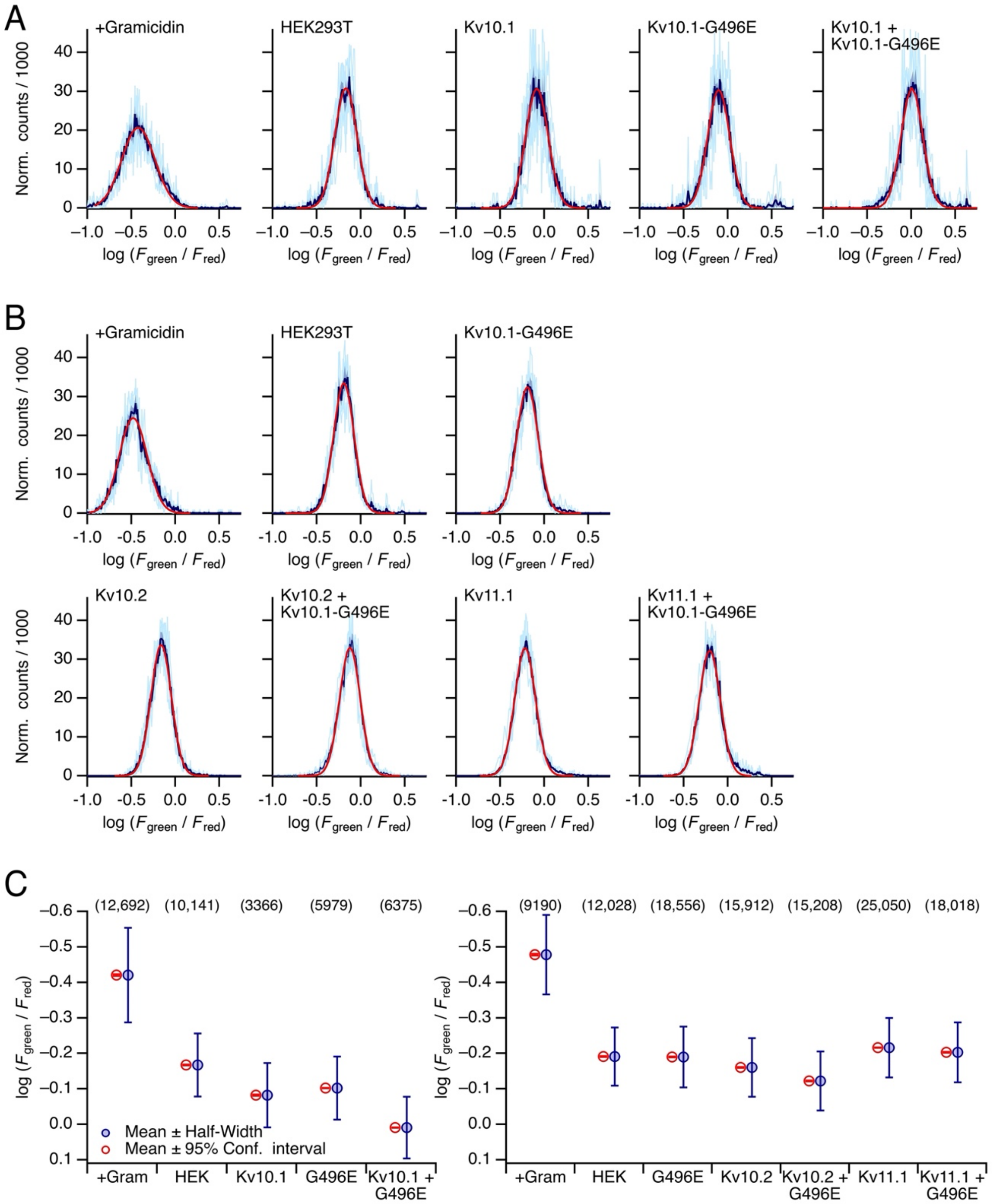

Supplementary Figure 6 Distributions of the ratiometric mK2-rEstus fluorescence in HEK293T cells (raw data set for the results shown in Fig. 4). **A, B**) Normalized histograms of log(F_{green} / F_{red}) from HEK293T cells expressing mK2-rEstus with the indicated treatments or expressions for two different sets of experiments corresponding to the data shown in Fig. 4A and Fig. 4B, respectively. The light blue traces are histograms based on individual cell culture dishes (N = 6); the dark blue traces are the means of the individual histograms, and the red curves are the results of fitting a Gaussian function to the averaged histograms. **C**) Results from the analysis shown in A) (*left*) and B) (*right*). The red symbols are the means of the Gaussian distributions ± 95% confidence interval (smaller than the symbols); the blue symbols are the means of the distributions ± the half-width of the fitted distributions. Note that the half-width is largest for cells treated with gramicidin because at 0 mV the

fluorescence intensity is smaller than in all other polarized cases. The total number of cells analyzed is given in parentheses.

References

1. Whicher JR, MacKinnon R. Structure of the voltage-gated K^+ channel Eag1 reveals an alternative voltage sensing mechanism. Science. 2016;353(6300):664-9.
